# Detecting and managing hypertensive disorders in pregnancy: a cross-sectional analysis of the quality of antenatal care in Nigeria

**DOI:** 10.1186/s12913-019-4217-8

**Published:** 2019-06-24

**Authors:** Angela Salomon, Salisu Ishaku, Karen R. Kirk, Charlotte E. Warren

**Affiliations:** 1 Dalla Lana School of Public Health, Toronto, Canada; 2 Population Council, Abuja, Nigeria; 3 Population Council, New York, USA; 4 Population Council, Washington, USA

**Keywords:** Antenatal care - maternal health - quality of care - health systems, Hypertensive disorders in pregnancy - primary health care

## Abstract

**Background:**

Nigeria has one of the highest rates of maternal mortality in the world (576/100,000 births), with a significant proportion of death attributed to hypertensive disorders in pregnancy (HDPs). High quality antenatal care (ANC) plays a crucial role in early detection and management of HDPs. We conducted an assessment of quality of antenatal care, and its capacity to detect and manage HDPs, in two tiers of Nigerian facilities, with the aim of describing the state of service delivery and identifying the most urgent gaps.

**Methods:**

Quality of antenatal care was assessed and compared between primary healthcare centers (PHCs) (*n* = 56) and hospitals (secondary + tertiary facilities, *n* = 39) in seven states of Nigeria. A cross-sectional design captured quality of care using facility inventory checklists, semi-structured interviews with healthcare providers and clients, and observations of ANC consultations. A quality of care framework and scoring system was established based on aspects of structure, process, and outcome. Average scores were compared using independent sample t-tests and measures of effect were assessed by multivariate linear regression.

**Results:**

All domains of quality except provider interpersonal skills scored below 55%. The lowest overall scores were observed in provider knowledge (49.9%) and provider technical skill (47.7%). PHCs performed significantly worse than hospitals in all elements of quality except for provider interpersonal skills. Provider knowledge was significantly associated with their level of designation (i.e., obstetrician vs. other providers).

**Conclusions:**

In order to provide high quality care, ANC in Nigeria must experience massive improvements to inventory, infrastructure and provider knowledge and training. In particular, ANC programs in PHCs must be revitalized to minimize the disparity in quality of care provided between PHCs and hospitals. The relatively low quality of care observed may be contributing to Nigeria’s high rate of maternal mortality and burden of disease attributed to HDPs.

**Electronic supplementary material:**

The online version of this article (10.1186/s12913-019-4217-8) contains supplementary material, which is available to authorized users.

## Background

Antenatal care (ANC) describes the services offered to pregnant women, including health promotion and communication, screening and diagnosis, disease prevention, and emotional and psychological support. Quality, evidence-based ANC plays a crucial role in improving the lives of pregnant women, and in setting the groundwork for healthy motherhood and infant development [1]. As of 2015, hypertensive disorders in pregnancy (HDPs), including pre-eclampsia and eclampsia, were the greatest cause of facility-based maternal mortality (29.0%) in Nigeria [2]. Broadly speaking, HDPs are characterized by elevated blood pressure, proteinuria, and many hematologic, hepatic, neurologic and renal changes that can result in adverse maternal and neonatal outcomes including intrauterine growth restriction, oligohydramnios (low amniotic fluid), placental abruption, and fetal death [3]. Because HDPs tend to have easily detectable clinical parameters [4], increasing utilization of quality ANC is essential to reducing maternal mortality caused by this class of disease.

Globally, an estimated 81% of pregnant women attend at least one antenatal care visit, while only 56% attend at least four [5]. In regions of sub-Saharan Africa, this number is further reduced [6], and in Nigeria specifically, only half of pregnant women attend at least four visits, ranging from 30% in north-western states to 87% in the southwest [7, 8]. While increasing access to ANC services is important, it is also critical that the services received are of good quality. Quality of Care (QOC), as described by Donabedian [9], depends on three components: structure (adequacy of physical environment and systems), process (components of care delivered), and outcomes (satisfaction/status of clients) [9, 10]. That is to say, facilities should be well stocked with essential commodities, services should be provided by competent healthcare workers, and clients should leave well-informed, satisfied, and respected.

In Nigeria, primary healthcare centers (PHCs) are the first points of contact for many pregnant women, especially for the majority of rural-dwellers; however, service readiness in PHCs is often sub-standard compared to hospital counterparts [11, 12]. Recently, a landscape analysis of pre-eclampsia and eclampsia in Nigeria [13] showed limited capacity at PHCs for providing even the most basic emergency obstetric and neonatal care. Previous research has explored factors affecting quality of ANC in Nigeria but has been restricted to private health facilities and small geographic areas and has used a limited scope of quality of care [14–16]. This paper explores the quality of ANC in seven states across the six geo-political zones of Nigeria using a well-established framework for assessing QOC, with a focus on its capacity to detect and manage hypertensive disorders in pregnancy. Furthermore, it examines disparities in the quality of ANC provided between PHCs and hospitals.

## Methods

### Study participants

**Fig. 1:**
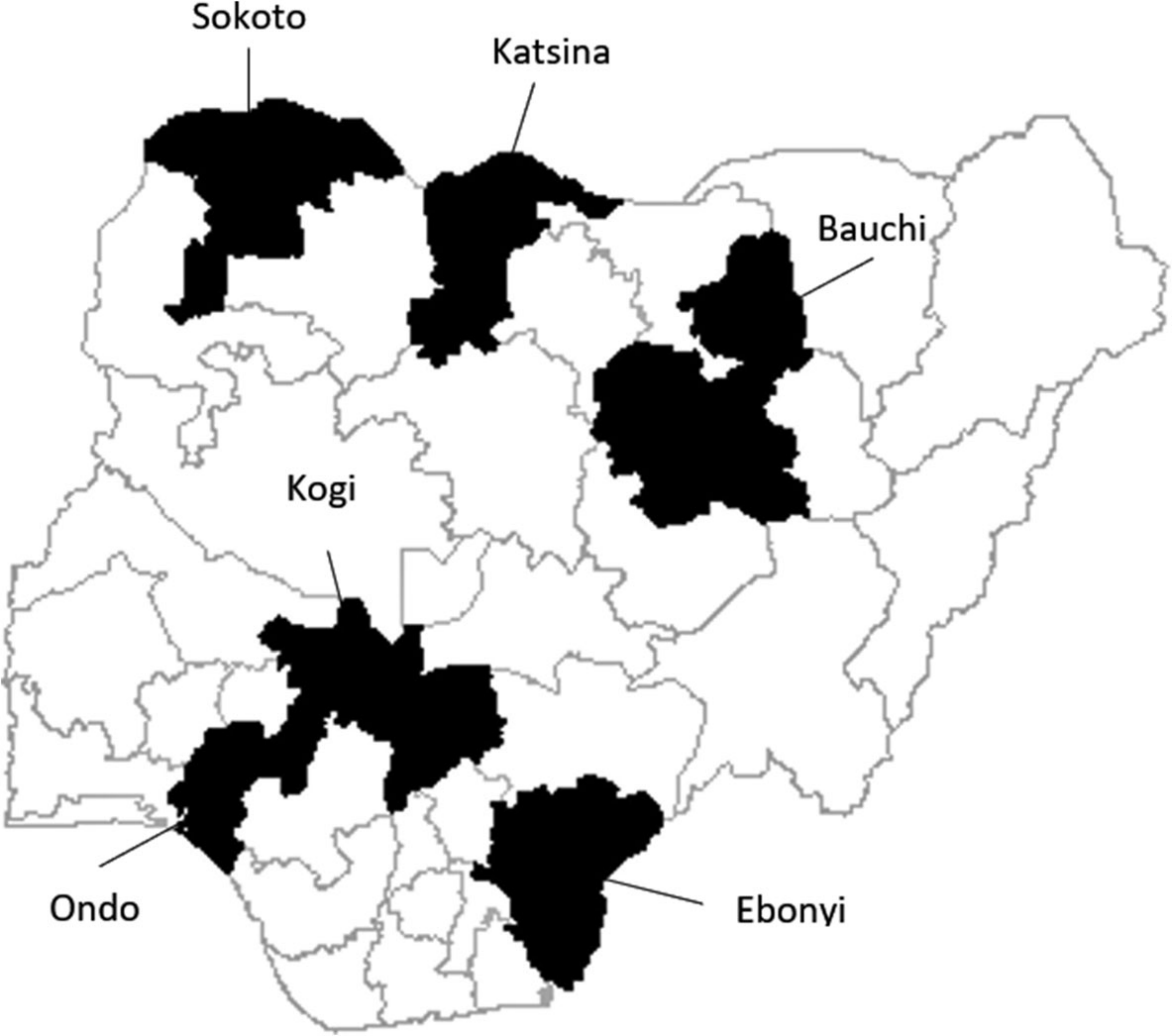
Map of study sites. Data was collected from 95 participating hospitals in 7 states across the 6 geopolitical zones of Nigeria. Geopolitical zones are divided based on similarity in culture, ethnic group, and common history. Including all hospitals from all geopolitical zones helps to ensure representative national results. Rights to this image are owned by the author

Following discussion with the Nigerian Federal Ministry of Health and the donor, seven states covering the country’s six geo-political zones were chosen as study sites – Sokoto, Bauchi, Katsina, Kogi, Cross River, Ebonyi, and Ondo (Fig. 1). In consultation with the states’ Ministries of Health, ninety-five public facilities with ANC units were purposively selected from within these states to represent diversity in facility type (PHCs = 56, secondary/tertiary hospitals = 39). Following an inventory assessment at 95 selected facilities, researchers conducted service provider interviews (*n* = 200) from a subset of 93 facilities; 2 facilities were excluded as no willing providers were present on the day of the inventory assessment. Researchers also observed 135 ANC consultations and conducted 135 client exit interviews from a subset of 26 facilities. The subset of facilities used for consultation observations and client interviews was chosen using convenience sampling, based on the location of the facility (considering difficult terrains), as well as the functionality of the facility (facility operating hours), while ensuring a representative sample across facility level and region.

### Quality of Care framework

The framework used for measuring quality of ANC was adapted from Warren et al. [17] and included many of the essential elements of antenatal care as informed by WHO guidelines [1]. It was further tailored to include indicators specifically pertaining to the capacity of the ANC to detect and manage HDPs.

The framework incorporated three elements of QOC as described by Donabedian [9], Bruce [18], and Hulton [19]: structure, process, and outcome. **Structure** encompassed factors related to inventory and infrastructure (facility guidelines, equipment, supplies, drugs, referral mechanisms, etc.), as well as human resources (provider knowledge and training). **Process** encompassed the range of services provided, information and documentation shared with the client, the practitioner’s competency in history taking and physical examinations, and their interpersonal skills. **Outcome** encompassed client experiences, satisfaction, and health comprehension. Table 1 shows the indicators used to create structure, process, and outcome scores. Scores were calculated as the average cumulative number of points received by each unit of observation (facility, provider, ANC consultation, or client interview). All scores are reported as a percent of the total possible score.

**Table 1:** Attributes of care: structure, process and outcome

Attributes of quality	Indicators
Structure attributes: Infrastructure equipment and supplies (0–38) *Data source: facility inventory*	
Data collection tools (0–3)	Registers for: ANC visits, inpatients and referrals, maternal deaths
Infrastructure (0–6)	Source of clean water, 24 h power for fridge, autoclave/sterilization, lighting, infection prevention buckets, chlorine for processing equipment
PE/E Guidelines (0–5)	Protocols for diagnosis PE/E, managing PE/E, administering MgSO_4_
General Equipment (0–11)	Stethoscopes, sphygmomanometers, urine collection containers, dipsticks, patella hammers, self-retaining catheter, urine bag, 20 ml syringe, saline (IV), IV equipment, syringe/needles
Availability of Drugs (0–5)	Availability of magnesium sulfate, calcium gluconate, anti-hypertensives, xylocaine, misoprostol
Referral Mechanisms (0–4)	Transport system/ambulance, 24/7 referral system, facility makes transport arrangements, client is accompanied to referral facility
Capacity for HDP-specific services (0–4)	See pre-eclamptic/eclamptic patients in ANC visits, admit pre-eclamptic patients into labor ward, admit clients with eclampsia to maternity ward
Structure attributes: provider knowledge and training (0–66) *Data source: provider interview*	
Trainings in past 3 years (0–7)	Any maternal/FP training, emergency obstetric care, ANC, safe delivery, skilled birth attendant, family planning, lab investigations
Signs and Symptoms of HDPs (0–12)	Definition of HDPs, signs of mild PE (Y/N), signs of eclampsia (Y/N), signs of severe PE: high BP, proteinuria, severe headaches, changes in vision, light sensitivity, upper abdominal pain, nausea/vomiting, decreased urine output, edema
Management/Treatment of PE (0–8)	Admit, start monitoring signs, monitor BP, monitor fetal heart rate, monitor fluid input/output, quantitative monitoring of proteinuria, administer anti-hypertensive, refer to nearest doctor/specialist
Management of Severe PE (0–9)	Admit, start monitoring signs, monitor BP, monitor fetal heart rate, monitor fluid input/output, quantitative monitoring of proteinuria, administer anti-hypertensive, administer anti-seizure/magnesium sulfate, refer to nearest doctor/specialist
Management of Eclampsia (0–11)	Admit, start monitoring signs, monitor BP, monitor fetal heart rate, monitor fluid input/output, quantitative monitoring of proteinuria, keep tongue blade ready for possible seizure, administer anti-hypertensive, administer anti-seizure/magnesium sulfate, induce delivery, refer to nearest doctor/specialist
Diagnosis of HDPs (0–3)	Chronic hypertension, PE, eclampsia
Use of MgSO_4_ (0–7)	Know total loading dose for MgSO_4_, know maintenance dose for MgSO_4_, 2 ways to monitor toxicity, drug used to treat toxicity, how to administer drug to treat toxicity, 1 advantage/disadvantage of using MgSO_4_, believe that morbidity/mortality will decrease with use of loading dose
Eclampsia Prophylaxis (0–4)	Guidelines on giving prophylaxis to prevent PE, which drug to give to prevent PE, currently use aspirin among women at risk of PE/E, 1 advantage/disadvantage of using prophylaxis to prevent PE
Antihypertensive Drugs (0–5)	Use antihypertensive to control HT in PE patients, use vasodilators to control severe HT in PE/E patients, know correct BP at which anti-hypertensives are administered, know correct BP at which vasodilators are administered, use antihypertensive to treat pregnant women with high BP
Process attributes: provider technical skills (0–37) *Data source: client-provider observations and client exit interview*	
History taking (0–9)	Date of last menstrual period, any current medication, general health problems, history of hypertension/high BP, diabetes, asthma, TB, malaria, STI/STD
Previous pregnancy history taking (0–6)	Type and duration of last delivery, history of miscarriage/abortion, stillbirth, neonatal death, cesarean section
Physical examination (0–9)	Weight, blood pressure, pulse, conjunctiva, body temperature, edema, abdomen palpation, fetal movement/heart rate
Lab tests (0–2)	Urine: glucose, albumin
Health promotion/ disease prevention (0–12)	Provided: malaria prophylaxis, tetanus toxoid injection, iron/folic acid supplements, HIV test; took blood sample, urine sample; gave advice/information on: diet & nutrition, insecticide-treated bed nets, STIs/AIDS, PMTCT of HIV, danger signs of pregnancy, self-care
Encouragement of continuation of care (0–3)	Told when and where to return for follow-up, given written reminder of date for follow-up
Documentation (0–2)	nfo recorded on: client’s ANC card, in registers
Process attributes: provider interpersonal skills (0–11) *Data source: client-provider interactions*	
Rapport (0–7)	Used client’s name, greeted client in friendly manner, requested client to take a seat, maintained audio & visual privacy, treated client with respect, client felt comfortable asking questions
Communication (0–4)	Used words client could understand, listened to client attentively, inquired for need of other services, answer client’s inquiries
Outcome attributes: client experiences (0–16) *Data source: client exit interview*	
Wait and cost (0–3)	Time spent waiting was reasonable, time with provider was reasonable, amount paid was reasonable
Satisfaction (0–5)	Satisfied with services received, would recommend to a friend, will return: before giving birth, 1 week after birth, 6 weeks after birth
Health comprehension/literacy (0–8)	Opportunity to ask questions, encouraged to return for another visit, discussed progress of pregnancy, provided results from: checkup, blood pressure, urine test; explained results from: blood pressure, urine test
Categorical and Continuous Outcome Measures	
Wait time (average)	Wait before seeing provider
Time spent with provider (average)	Length of consultation
First ANC Visit	Proportion of women attending a first ANC visit in < 10 weeks, 10–19 weeks, 20–29 weeks, or 30+ weeks
Amount willing to pay	Amount client is willing to pay for services (in USD)
Satisfaction	Proportion of women satisfied, somewhat satisfied, or not at all satisfied with services
Knowledge of danger signs	Proportion of women who know none, 1, 2, or 3 danger signs of pregnancy that would make them return to the facility

### Data collection

This paper uses cross sectional data collected during a landscape analysis that assessed the health facility and provider capacity to prevent, detect, and manage preeclampsia and eclampsia in seven states in Nigeria [13]. Data collection occurred over a three-month period, from June–August 2015.

#### Structure

Inventory and infrastructure (*n* = 95) were assessed with a facility inventory checklist for a maximum score of 38 points. Provider knowledge, competence, and training was assessed using semi-structured interviews with the ANC service providers (*n* = 200) for a maximum score of 66 points (Table 1).

#### Process

Client-provider interactions (*n* = 135) were observed and documented using a standardized checklist to assess the provider’s technical skills for a maximum score out of 37, and the provider’s interpersonal skills for a maximum score out of 11 (Table 1).

#### Outcome

Following their ANC visit, researchers conducted semi-structured exit interviews with clients (n = 135) to assess their perception, satisfaction, and knowledge of services received, for a maximum score out of 16 (Table 1).

### Data analysis

We compared mean scores obtained from PHCs and hospitals in each QOC category using one-tailed independent sample t-tests (H_0_: secondary facility = PHCs, H_A_: secondary facility > PHC); variances were assumed unequal if Levene’s Test for Equality was significant. For all categorical variables, significant differences between facility type were determined using the Pearson chi-square test; Fisher’s Exact test was used in cases where individual cell counts were below 5.

We then conducted two multivariate linear regression models to identify independent variables associated with provider knowledge and outcome scores. Variables in model 1 included facility level, provider age, gender, and designation; variables in model 2 included facility level, client age, education, and socio-economic status (SES). Clustering within facility was adjusted for using a robust estimate of variance (sandwich estimator).

We developed a single variable for SES through principal component analysis of indicators pertaining to wealth, household facilities, and assets (including vehicles, land, and livestock owned). We classified SES as either low, medium or high based on whether the individual fell into the bottom, middle, or top tertile [20].

A significance threshold of *p* = 0.05 was used for all analyses. Individual observations with > 50% of data missing were excluded from analysis. Where appropriate, means (with standard deviations), coefficients (with 95% confidence intervals), and *p*-values are reported. Data were stored, cleaned, and analyzed using STATA v 15.1 [21]

## Results

**Fig. 2:**
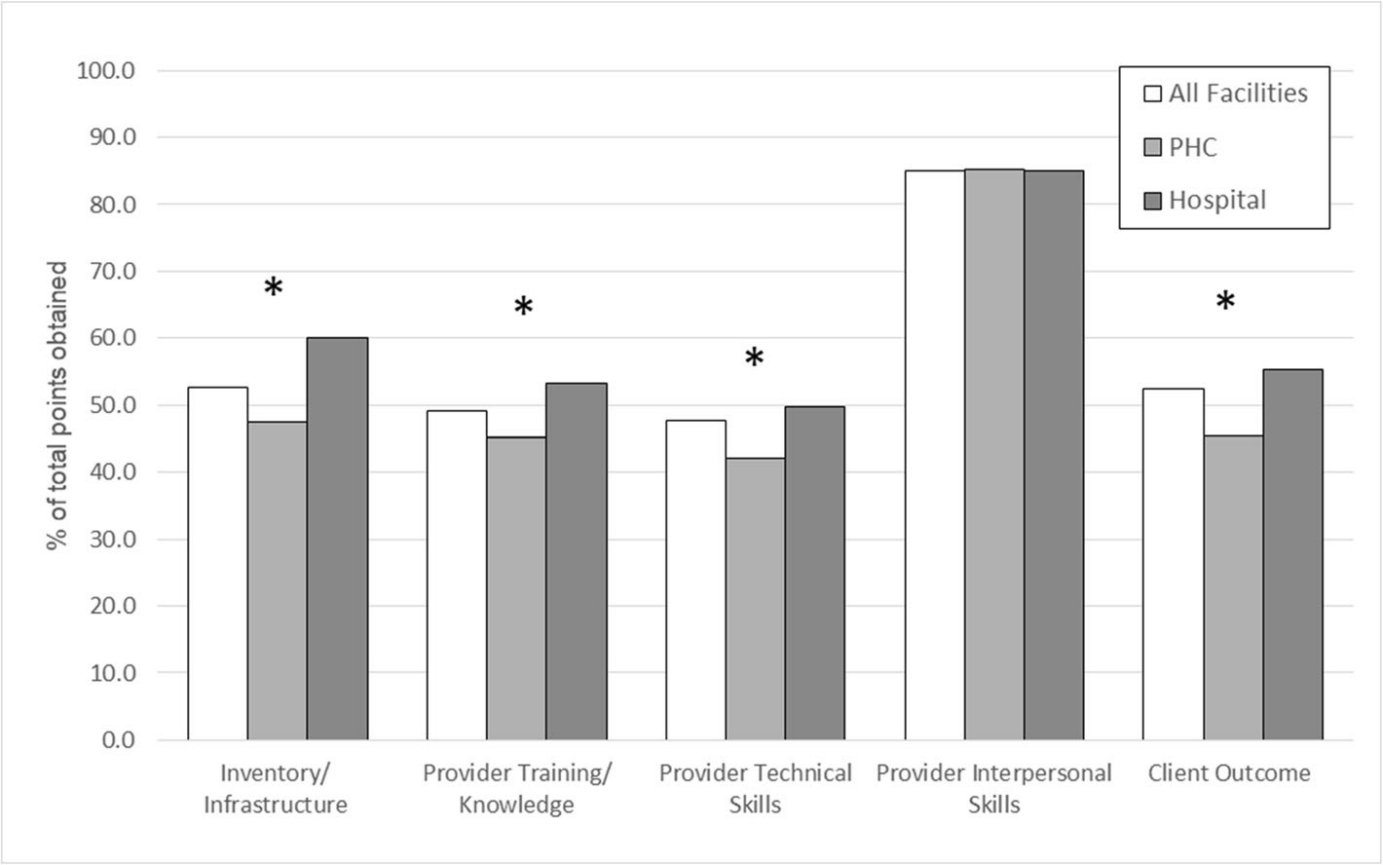
Comparison of domains of quality of care across facility type**.** All domains except for interpersonal skills obtained a cumulative score of below 55%. The lowest overall scores were obtained in provider and provider skill (47%) and provider training/knowledge (49%), while the highest scores were obtained in provider interpersonal skills (85%). PHCs scored lower than hospitals in all domains except provider interpersonal skills

Figure 2 shows an overview of the scores received in each of the five categories by all facilities, PHCs, and Hospitals.

### Descriptive characteristics

Client and provider characteristics are described in Table 2. The average client was 27.78 years of age, with those visiting PHCs being significantly younger than those visiting hospitals (24.9 vs. 28.3 years; *p* = 0.001). Most women had completed at least secondary level of schooling (30.1%), and the level of education attained was significantly associated with facility type, with a much higher percentage of women at hospitals having attended postsecondary school (*p* < 0.001). Those identifying as Muslim were approximately three times more likely to attend a PHC than a hospital facility (p = 0.001), likely because the majority of PHCs sampled were in Northern states, which are predominantly Muslim. There were no significant differences between PHCs and hospitals in the client’s SES, marital status, or spending decision habits.

The mean age of providers (39.1 years) was similar at both PHCs and hospitals, and the majority were female (81.6%). In PHCs, most providers were Community Health Extension Workers (CHEWs) (64.8%), followed by nurse/midwives (18.1%); this was significantly different from hospitals, where the majority of provides were nurses/midwives (66.7%), followed by general practitioners (15.6%) (*p* < 0.001).

**Table 2:** Characteristics of study clients and providers across facility type

Client characteristics	Total	PHC	Hospital	*P*-Value
	(*n* = 135)	(*n* = 38)	(*n* = 97)	
Mean age (years) ± SD	27.87 ± 8.23	24.89 ± 5.88	28.32 ± 5.15	0.001
Mean gravidity ± SD	2.79 ± 2.05	3.16 ± 2.49	2.65 ± 1.84	0.256
Mean gestational age (weeks) ± SD	28.26 ± 8.43	30.13 ± 8.56	27.43 ± 8.29	0.120
Mean gestational age at 1st ANC visit (weeks) ± SD	20.83 ± 7.78	21.9 ± 8.06	20.49 ± 7.71	0.413
Mean # ANC visits^b^				
Gestational age < 20 weeks (*n* = 13)	2.23 ± 1.79	2.00 ± 2.00	2.33 ± 1.80	0.771
Gestational Age 20–29 weeks (*n* = 27)	2.15 ± 1.54	2.60 ± 1.52	2.05 ± 1.56	0.477
Gestational age 30+ weeks (*n* = 64)	3.43 ± 2.09	3.39 ± 1.9	3.45 ± 2.21	0.920
Education [n (%)]				<0.001
Primary or below primary	41 (30.4)	18 (47.4)	23 (23.7)	
Secondary	41 (30.1)	19 (50)	22 (22.7)	
Postsecondary	53 (39.3)	1 (2.6)	52 (53.6)	
Religion [n (%)]				0.001
Christian	60 (44.4)	8 (21.1)	52 (53.6)	
Muslim	75 (55.6)	30 (78.9)	45 (46.4)	
Marital status [n (%)]				1.000^a^
Married/living together	130 (96.3)	37 (97.4)	93 (95.9)	
Divorced/separated/widowed	5 (3.7)	1 (2.6)	4 (4.1)	
Health insurance coverage [n (%)]^b^				0.191
Yes	19 (23.8)	2 (11.8)	17 (27.0)	
No	61 (76.3)	15 (88.2)	46 (73.0)	
Socioeconomic status [n (%)]				0.051
Low	50 (37.0)	9 (23.7)	41 (42.3)	
Medium	63 (46.7)	24 (63.2)	39 (40.2)	
High	22 (16.3)	5 (13.2)	17 (17.5)	
Spending decision [n (%)] ^b^				0.285
Myself	10 (8.3)	3 (8.6)	7 (8.2)	
Partner	49 (40.8)	16 (45.7)	33 (38.8)	
Both myself and partner	51 (42.5)	11 (31.4)	40 (47.1)	
Other	10 (8.3)	5 (14.3)	5 (5.9)	
PROVIDERS	Total	PHC	Hospital	
	(*n* = 201)	(*n* = 105)	(*n* = 96)	
Mean age ± SD	39.06 ± 9.64	38.15 ± 9.79	40.10 ± 9.40	0.164
gender [n (%)]				0.396
Male	37 (18.4)	17 (16.2)	20 (20.8)	
Female	164 (81.6)	88 (83.8)	76 (79.2)	
Type of provider [n (%)]				<0.001
Obstetrician/Gynecologist	6 (3.0)	0 (0.0)	6 (6.3)	
General Practitioner	20 (10.0)	5 (4.8)	15 (15.6)	
Nurse/midwife	83 (41.3)	19 (18.1)	64 (66.7)	
Community Health Extension Worker	79 (39.3)	68 (64.8)	11 (11.5)	
Community Health Officer	13 (6.5)	13 (12.4)	0 (0.0)	

^a^ *using Fisher’s exact test*

^b^ *effective sample size does not equal total sample due to missing data*

### Structural attributes of care: inventory/infrastructure

Table 3 shows the structural attributes related to facility inventory and infrastructure. Out of a total of 38 points, PHCs scored significantly lower than hospitals (47.5% vs. 60.0%, p < 0.001). PHCs also scored significantly lower in domains of general infrastructure (40.8% vs. 54.3%; *p* = 0.0116), general equipment (54.2% vs. 64.6%, *p* = 0.0304), referral mechanisms (54.9% vs. 67.3%; *p* = 0.0161) and capacity for HDP specific services (38.8% vs. 68.6%; *p* < 0.0001). There were no significant differences between facility type in scores for data collection tools (90.5%), HDP guidelines (33.9%), or availability of drugs associated with HDPs (38.5%).

**Table 3:** Average Scores for Structural Attributes

Structure Attributes	Total			Primary			Secondary			*p*-value
	Mean	SD	% of total	Mean	SD	% of total	Mean	SD	% of total	
Data collection tools (0–3)	2.72	0.56	90.5	2.64	0.59	88.1	2.82	0.51	94.0	0.059*
Infrastructure (0–6)	2.78	0.18	46.3	2.45	0.21	40.8	3.26	0.29	54.3	0.012
Guidelines (0–5)	1.69	0.20	33.9	1.45	0.25	28.9	2.05	0.33	41.0	0.071
General equipment (0–11)	6.43	0.30	58.5	5.96	0.40	54.2	7.10	0.43	64.6	0.030
Drugs (0–5)	1.93	0.12	38.5	1.79	0.14	35.7	2.13	0.22	42.6	0.087
Referral mechanisms (0–4)	2.40	0.11	60.0	2.20	0.14	54.9	2.69	0.18	67.3	0.016
Capacity for HDP-specific services (0–4)	2.04	0.14	51.1	1.55	0.16	38.8	2.74	0.19	68.6	0.000
**Total for inventory/ infrastructure (0–38)**	**19.99**	**0.73**	**52.6**	**18.04**	**0.91**	**47.5**	**22.79**	**1.06**	**60.0**	**0.001**
Training in last 3 years (0–6)	1.18	0.10	19.7	1.13	0.13	18.8	1.24	0.17	20.7	0.293
Signs of HDPs (0–12)	7.66	0.22	63.8	7.21	0.32	60.1	8.14	0.30	67.8	0.019
Management of pre-eclampsia (0–8)	4.15	0.18	51.8	4.22	0.25	52.8	4.06	0.26	50.8	0.669
Management of severe pre-eclampsia (0–9)	4.85	0.24	53.9	4.42	0.35	49.2	5.31	0.33	59.0	0.032*
Management of eclampsia (0–11)	6.29	0.32	57.1	5.47	0.46	49.7	7.17	0.44	65.2	0.004
Diagnosis of HDPs (0–3)	1.97	0.07	65.5	1.74	0.10	58.0	2.21	0.09	73.6	0.000
Usage of MgSO4 (0–7)	1.63	0.12	23.2	1.12	0.13	15.9	2.18	0.20	31.1	0.000*
Usage of eclampsia prophylaxis (0–4)	3.81	0.04	95.1	3.89	0.05	97.4	3.71	0.06	92.7	0.994*
Usage of antihypertensive drugs (0–5)	0.92	0.08	18.4	0.68	0.09	13.7	1.18	0.13	23.5	0.001*
**Total knowledge/ training score (0–65)**	**32.44**	**0.92**	**49.9**	**29.88**	**1.25**	**46.0**	**35.19**	**1.31**	**54.1**	**0.002**

*equal variances not assumed (using F statistic, p < 0.05)

### Structural attributes of care: provider knowledge/training

Table 3 shows the structural attributes related to provider knowledge and training. Out of a total of 66 points, providers in PHCs scored significantly lower than those in hospitals (46.0% vs. 54.1%, *p* = 0.002). PHCs scored lower than hospitals in additional facets of provider knowledge including signs and symptoms of HDPs (60.1% vs. 67.8%; *p* = 0.019), how to manage severe pre-eclampsia (49.2% vs. 59.0%; *p* = 0.032) and eclampsia (49.7% vs. 65.2%), correctly diagnosing HDPs (58.0% vs. 73.6%; *p* = 0.000), correct use of magnesium sulfate (15.9% vs. 31.1%, p = 0.000), and use of antihypertensive drugs to manage mild HDP (13.6% vs. 23.5%, *p* = 0.001).

**Fig. 3:**
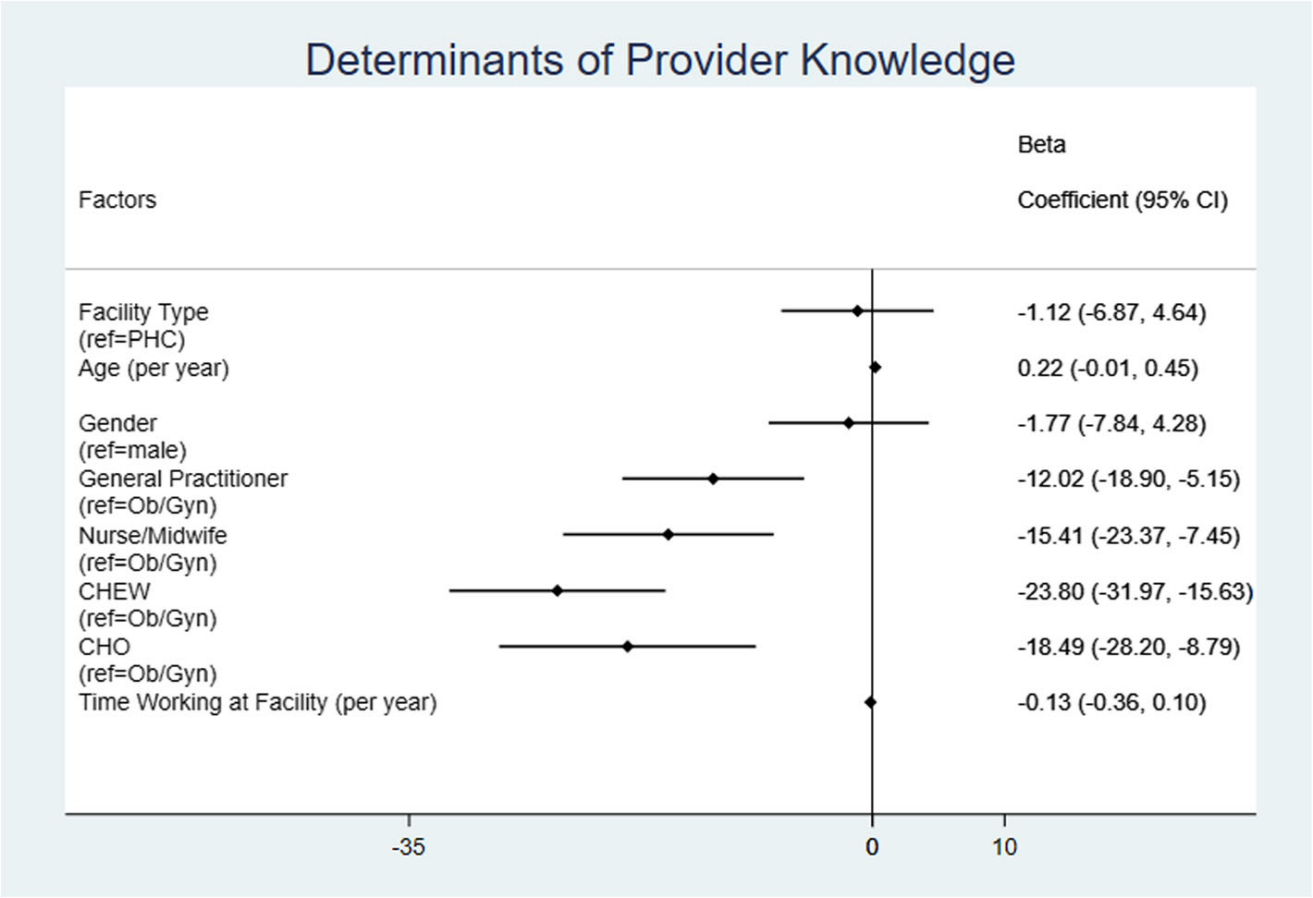
Linear regression coefficients examining provider knowledge score**.** Each row represents a variable included in the multivariable linear regression assessing factors associated provider knowledge scores. After adjustment for provider sociodemographic factors, the level of facility (primary vs. secondary) was no longer significant. Compared to obstetricians/gynecologists (reference group), each of the other provider types (general practitioner, nurse/midwife, CHEW, and CHO) obtained significantly lower knowledge scores

The difference observed in overall provider knowledge between PHCs and hospital facilities was no longer significant after regression adjustment for provider demographic variables including age, gender, type of provider, and length of time working at facility (Fig. 3). However, overall provider knowledge was significantly associated with provider type; using maternal health specialists as the reference (obstetricians/gynecologists), it was found that general practitioners, nurses/midwives, CHEWs, and CHOs scored lower by 12.0 points (p = 0.001), 15.4 points (*p* = 0.000), 23.8 points (p = 0.000) and 18.5 points (p = 0.000), respectively. Further adjustment for clustering by specific facility did not quantitatively or qualitatively change the results of the regression analysis.

### Process attributes of care: provider technical skills

Table 4 shows the process attributes related to provider technical skills. Out of a total of 37 points, PHCs scored significantly lower than hospitals (42.2% vs. 49.8%, *p* = 0.0017). There were also significant differences between facility type for skills in general history taking (42.2% vs. 61.0%; *p* < 0.001) and history taking for women with a previous pregnancy (39.6% vs. 58.8%; *p* = 0.0073). There were no significant differences in scores for physical examinations, lab tests, documentation, health promotion/prevention of disease, or encouragement of follow-up, but all categories except for documentation and encouragement of follow-up scored below 70% of potential points.

**Table 4:** Average Scores for Process Attributes

Process Attribute	Total			Primary			Secondary			*p*-value
	Mean	SD	% of total	Mean	SD	% of total	Mean	SD	% of total	
History taking for women with previous pregnancy (0–6)	3.22	0.22	53.7	2.38	0.45	39.6	3.53	0.25	58.8	0.011
History taking (0–9)	5.00	0.23	55.6	3.79	0.49	42.2	5.49	0.24	61.0	0.000
Physical exam (0–9)	5.64	0.13	62.6	5.62	0.18	62.4	5.65	0.16	62.7	0.457
Lab tests (0–2)	1.04	0.08	52.2	0.85	0.16	42.3	1.13	0.09	56.3	0.060
Documentation (0–2)	1.46	0.06	73.0	1.31	0.12	65.4	1.52	0.07	76.0	0.063
Disease prevention (0–12)	8.35	0.21	69.6	8.47	0.34	70.6	8.30	0.26	69.2	0.644
Follow-up/continuation of care (0–3)	2.35	0.08	78.3	2.44	0.12	81.2	2.31	0.10	77.1	0.752
Total provider/technical skills score (0–37) ^a^	17.64	0.44	47.7	15.61	0.84	42.2	18.43	0.49	49.8	0.002
Rapport (0–7)	6.21	0.10	88.7	6.23	0.16	89.0	6.20	0.13	88.5	0.559
Communication (0–4)	3.15	0.07	53.7	3.15	0.11	78.8	3.15	0.08	78.6	0.522
**Total interpersonal skills score (0–11)**	**9.36**	**0.137**	**85.1**	**9.38**	**0.21**	**85.3**	**9.34**	**0.17**	**84.9**	**0.553**

^*a*^ *excluding history for women with previous pregnancy, as this category is relevant only for subset of population*

### Process attributes of care: provider interpersonal skills

Table 4 also shows the process attributes related to provider interpersonal skills. Out of a total score of 11, PHCs and hospitals scored highly – 85.3 and 84.9%, respectively. There were no significant differences observed in the scores of interpersonal sub-categories, or in the cumulative interpersonal score between PHC and hospital care providers. However, individual indicators within the rapport-building and communications sub-categories were found to significantly differ (see Additional file 1). For example, PHCs more often used the client’s name (97.5% vs. 89.5%; *p* = 0.016) and greeted in a friendly manner (100% vs. 85.4%; *p* = 0.011), but were less likely to maintain audio privacy (67.5% vs. 92.6%; *p* < 0.001) and visual privacy (65.0% vs. 90.5%; p < 0.001), or inquire for need of other services (35.9% vs. 83.3%; *p* = 0.022).

### Outcome attributes of care

Table 5 shows the outcome attributes related to client experiences. Out of a total of 16, PHCs scored significantly lower than hospitals (45.4% vs. 55.2%; *p* = 0.004). While perceptions of wait times, costs, and satisfaction were not significantly different between facility type, clients at PHCs had significantly lower health literacy than those at hospital health facilities (39.5% vs. 50.6%, *p* = 0.020). Wait-time and time spent with providers were comparable between primary and secondary facilities, but women attending ANC at hospitals reported willingness to pay a higher amount for services (1.28 USD vs. 0.43 USD; *p* = 0.044) (Table 6). More than half of all women (51.1%) could not recall any danger signs in pregnancy that would compel them to return to a health provider. Only 5.2% of clients could name at least 3 danger signs – most commonly severe headache, bleeding or fluids from vagina, and swelling of legs and feet (Table 6). Notably, satisfaction was very high for all facilities; 100% of respondents in PHCs and 89.4% of respondents in hospitals reported being satisfied with services received.

**Table 5:** Average Scores for Outcome Attributes

Outcome Attribute	Total			Primary			Secondary			*p*-value
	mean	SD	% of total	mean	SD	% of total	mean	SD	% of total	
Wait and cost (0–3)	1.58	0.09	52.6	1.34	0.17	44.7	1.67	0.11	55.7	0.052
Satisfaction (0–5)	3.01	0.11	60.3	2.76	0.23	55.3	3.11	0.12	62.3	0.092*
Health literacy (0–8)	3.80	0.20	47.5	3.16	0.33	39.5	4.05	0.24	50.6	0.020
**Total outcome score (0–16)**	**8.39**	**0.26**	**52.5**	**7.26**	**0.51**	**45.4**	**8.84**	**0.30**	**55.2**	**0.004**

*equal variances not assumed (using F statistic, p < 0.05)

**Table 6:** Categorical and Continuous Outcomes

Key outcome measures of quality of care	Total	PHC	Hospital	*p*-value
	(*n* = 135)	(*n* = 38)	(*n* = 97)	
Mean wait time (minutes) ± SD	93.8 **±** 78.13	96.41 **±** 81.0	92.85 **±** 77.63	0.839
Wait time [n (%)]^c^				0.285
<1 h	33 (31.4)	10 (37.0)	23 (29.5)	
1–2 h	45 (42.9)	8 (29.6)	37 (47.4)	
>2 h	27 (25.7)	9 (33.3)	18 (23.1)	
Mean time spent with provider (minutes) ± SD	20.4 ± 17.68	23.6 ± 19.8	18.8 ± 16.7	0.415
Time spent with provider [n (%)]^c^				0.185^a^
<5 min	9 (21.4)	1 (7.1)	8 (28.6)	
5–19 min	15 (35.7)	7 (50.0)	8 (28.6)	
20–39 min	10 (23.8)	2 (14.3)	8 (28.6)	
>40 min	8 (19.0)	4 (28.6)	4 (14.3)	
First ANC visit [n (%)]^c^				0.616^a^
<15 weeks	28 (24.1)	5 (17.9)	23 (26.1)	
15–24 weeks	41 (35.3)	9 (32.1)	32 (36.4)	
25–30 weeks	28 (24.1)	9 (32.1)	19 (21.6)	
>30 weeks	19 (16.4)	5 (17.9)	14 (15.9)	
Mean amount willing to pay for services (USD) ^b^ ± SD	1.06 ± 1.59	0.43 ± 0.40	1.28 ± 1.79	0.044
Amount willing to pay for services [n (%)] ^c^				0.741^a^
<0.27 USD	6 (19.4)	2 (25.0)	4 (17.4)	
0.27–1.37 USD	18 (58.1)	5 (62.5)	13 (56.5)	
>1.37 USD	7 (22.6)	1 (12.5)	6 (26.1)	
Satisfaction [n (%)]^c^				0.110^a^
Satisfied	107 (92.2)	31 (100.0)	76 (89.4)	
Somewhat or not satisfied	9 (7.8)	0 (0.0)	9 (10.6)	
Knowledge of danger signs in pregnancy [n (%)]				0.504^a^
0	69 (51.1)	21 (55.3)	48 (49.5)	
1	30 (22.2)	10 (26.3)	20 (20.6)	
2	29 (21.5)	5 (13.2)	24 (24.7)	
3	7 (5.2)	2 (5.3)	5 (5.2)	

^*a*^ *using Fisher’s exact test*

^*b*^ *1 USD = 364.50 NGN*

^*c*^ *effective sample size does not equal total sample due to missing data*

The difference observed in overall outcome score between PHCs and hospital facilities was no longer significant after regression adjustment for client age, level of education, and socioeconomic status (*p* = 0.189, [Additional file 2]). Outcome scores for women with a “high” SES were 1.42 (95% CI = 0.03, 2.80) points lower than scores for women with a “low” SES (*p* = 0.045). Further adjustment for clustering by specific facility did not quantitatively or qualitatively change the results of the regression analysis.

## Discussion

This analysis provides a comprehensive cross-sectional overview of the quality of ANC services provided across the six geo-political zones of Nigeria and explores how quality differs between PHC and hospitals. In general, it was found that the quality of ANC provided by Nigerian healthcare facilities is sub-par and does not meet many of the WHO-required elements for adequate ANC. All elements of quality of care, except for interpersonal skills, obtained a cumulative score of below 55%. Furthermore, PHCs scored lower than hospitals in most domains. Despite these relatively low scores for quality of care, self-reports of client satisfaction were very positive.

### Structure

Facilities captured in this study obtained very low scores for inventory and infrastructure - below half of the tools, recent guidelines, equipment, drugs, and mechanisms deemed necessary to perform antenatal services. Within general equipment, only three quarters of facilities had operational sphygmomanometers for measuring blood pressure, and less than half had dipsticks for proteinuria testing. These numbers are concerning, especially regarding the important role they play in diagnosing pre-eclampsia and eclampsia [22].

PHCs scored significantly lower than hospitals in almost all aspects of inventory and infrastructure. Ademiluyi and Aluko-Arowolo [23] have written on the unique infrastructural challenges faced by PHCs, citing Nigeria’s colonial history and its implications on the evolution and distribution of healthcare resources as the root cause. To this day, the bulk of public healthcare dollars are spent in secondary facilities, as is government spending on general infrastructure [24]. While all levels of facilities must meet standards for accreditation, secondary level facilities have much stricter infrastructure requirements, including those for operating theatre, pharmacy, laboratory, and personnel capacities [25]. Despite their limited capacity to treat pregnant women, PHCs play a vital role in advising women on danger signs of obstetric complications, detecting these complications (including pre-eclampsia and eclampsia) early and referring women to general or specialist hospitals in a timely manner (often following initial stabilizing care, such as administration of loading dose of MgSO_4_) [26]. However, PHCs likely face challenges in efficiently getting patients to hospitals in the case of emergencies, as this study reveals that only a quarter have any ambulatory transport systems.

Other research has found a similar lack of infrastructure in both primary and secondary facilities in Nigeria. Kress et al. [12] reported on a “general shortage of drugs and supplies” that likely are the result of segmented supply chains and financial constraints placed on Local Government Authorities (LGAs) who are responsible for allocating resources to PHCs. They report that as much as 95% of funding must be allocated to staff salaries at the expense of adequate drugs, supplies, and maintenance. Oyekale [11] similarly found that tools and drugs essential for ANC including sphygmomanometers, thermometers, stethoscopes, magnesium sulfate, folic acid, and calcium gluconate were unavailable or dysfunctional in as many as 87% of facilities studied.

Provider knowledge and training received the lowest overall scores, with PHCs scoring significantly lower than hospitals. This finding is not entirely unexpected given the presence of lower level staff at PHCs compared to secondary facilities. Indeed, regression analysis shows that provider knowledge scores are dependent on the type of provider, (with obstetricians/gynecologists scoring at least 12 points higher than the next best cadre – general practitioners). This is not to say that lower cadre health workers cannot be adequately trained on critical components of ANC, as has been demonstrated in previous research [27–30]. Despite existing evidence that shows task-shifting and training of lower-cadre healthcare workers is an important strategy for low-income countries, only 12.2% of providers in this study reported having received any training in the past 3 years.

### Process

For both PHCs and hospitals, scores for providers’ skills were moderate to low. Providers were relatively successful in the realm of ‘follow-up/ continuation of care’ (most clients were observed being instructed to come for a follow-up appointment and told where to go for follow-up). However, a smaller percentage were given a written reminder of *when* to come or encouraged to return in the case of emergency. It is well-documented that Nigerian women attend fewer ANC consultations than is recommended for healthy pregnancy and childbirth [8]. Providing written reminders may be a simple strategy to improve rates of ANC attendance. Previous research in Zanzibar has shown the effects of follow-up reminders (via SMS) to be beneficial in not only the number of ANC visits attended, but also in the percentage of women with antenatal complications identified and referred [31].

In 2015, Fagbamigbe and Idemudia [16] studied ANC quality in Nigeria and reported on the eight nationally recommended ‘critical components’ of ANC (blood pressure, iron supplements, blood sample, urine sample, tetanus injections, danger signs, HIV tests, malaria prophylaxis). As in our study, the most common of these eight components practiced were blood pressure measurement and provision of iron/folic acid supplements. Compared with those in Fagbamigbe and Idemudia, the participants in our study reported higher frequency of HIV testing (78.5% vs. 41.7%) and provision of malaria prophylaxis (77.8% vs. 40.1%), and lower frequency of urine testing (28.1% vs. 81.9%). Furthermore, process scores may be an optimistic interpretation of the provider’s skill in these areas; although the data tells us that the actions were taken, it does not specify the degree to which they were correctly completed. For example, there are many sources of inaccuracy for blood pressure measurement including patient, device, procedure, and observer-related sources [32]. Previous research has identified blood pressure inaccuracy as a challenge in hospitals in developing nations, and especially among providers with lower qualifications such as nurses and CHOs [33, 34].

Provider interpersonal skills, as assessed by direct observation of their interaction with clients, scored highly. Audio and visual privacy was the only interpersonal indicator in which PHCs performed worse than secondary facilities; this is understandable given the relatively little space and busy atmosphere of PHCs. Babatunde et al. [35] found similarly positive results among PHC users, with the ideal interpersonal skills being displayed in 71–91% of healthcare interactions.

### Outcome

Low scores in health literacy (dissemination, communication, and explanation of consultation results) were reflected in clients’ poor recall of danger signs of pregnancy. Hospitals did significantly better in this regard than PHC, possibly because of better-trained staff, or more educated clients. Gaining sufficient and adequate information about one’s health and potential complications from a care provider is important during the antenatal period and is associated with improved ANC attendance and pregnancy outcomes [36–38]. Health literacy is critical to patient empowerment, and accurate information from an informed provider is especially valuable when the alternative might be informal education, misrepresentation, or mythology.

Despite the low scores received for structural and process attributes, as well as moderate perceptions of wait time and cost, all facilities scored extremely well in terms of client satisfaction. This result is encouraging, as each new interaction with the health system presents an additional opportunity for HDPs to be detected and managed. However, perception of care must be interpreted cautiously, as low-income and low-education women can be uncritical of the healthcare they receive or base it on the provider’s interpersonal skills rather than their competence [17, 39, 40]. Only a quarter of clients attended an ANC visit before 15 weeks gestational age, despite the WHO’s recommendations for an initial contact within 12 weeks of pregnancy. This is especially important for first time mothers, and to ensure early detection of HDPs, HIV, and anemia [1].

In 2016, the WHO released “Standards for Improving Quality of Maternal and Newborn Care in Health Facilities” a document which outlines 8 domains of quality of care that should be assessed, improved, and monitored within the health system [41]. These range from the appropriate use of data, to effective and compassionate communication with women and their families, to appropriate physical environment and competent, motivated staff. Furthermore, quality antenatal care should result in a “positive pregnancy experience”, defined as not only maintaining a healthy pregnancy for the mother and baby, but “having an effective transition to positive labor and birth”, and “achieving positive motherhood (including maternal self-esteem, competence and autonomy)” [1]. In accordance with these standards, our study shows that improvements to ANC must be holistic and encompass not just the facility, but also provider knowledge and practices. It must also involve community mobilization to educate on the importance of ANC and improve maternal health literacy. In an environment where resources are limited and there are competing interests in terms of health priorities, solutions must be innovative and easy to adopt, especially for PHCs. This may include mobile training and decision-making tools, such as the m4Change application created as part of the Nigerian government’s Saving One Million Lives Initiative [29]. Policy that mandates regular training refreshers around ANC skills could help to close knowledge gaps. Improvements should be evidence-based and outcomes-oriented, meaning that they are measured using outcome indicators such as the number of women who have at least four ANC visits, tetanus protection at birth, or the proportion of women with a written birth and emergency plan at 37 weeks of pregnancy [42]. This research comes during the development of a new National Health Act, in which the Federal Government of Nigeria has committed to “reactivate” and “revitalize” the country’s PHCs. This has begun with the inauguration of an inter-agency Supply Chain Committee, which will act as the implementation partner for the PHC revitalization agenda [43]. This research may therefore serve as a framework for which major gaps in PHC quality, specifically as it pertains to ANC and the ability to manage HDPs, require immediate attention.

### Limitations

There are several limitations to our approach in the identification of indicators that adequately assess quality of ANC, and more specifically its capacity to detect and manage HDPs, as there is little consensus or predetermined framework for doing so. Quality and abundance of indicators are further limited in that they only include aspects of care that should be provided at *every* ANC consultation (data is cross-sectional and does not encompass multiple ANC visits for a single patient). Indicators used for “outcome” are limited in that they do not capture postnatal outcomes such as birth success, incidence of disease, survival, or long-term health conditions. However, they do measure outcomes of the consultation itself by showing how much knowledge and satisfaction a woman gained from her ANC visit, and how likely she would be to return. Facilities and clients were selected purposively, leading to potential selection bias and lack of external generalizability. However, because it was conducted across the country (and across all geo-political zones) – it reflects the population of most women attending ANC in Nigerian facilities. As with all studies that rely on interview-based data collection, this study is limited by interviewer and respondent bias. To mitigate this, research assistants were trained on interviewing and data collection techniques. Additionally, interview sources were supplemented with more objective data sources - facility and ANC visit observational checklists.

## Conclusion

We identified three major obstacles to quality ANC in Nigeria and its ability to detect and manage HDPs: 1) inadequate infrastructure, particularly in the lack of facility guidelines, drug stock, referral mechanisms, and general equipment including sphygmomanometers and urine dipsticks; 2) very inadequate provider knowledge, due to infrequent training updates and low understanding of the use of drugs to prevent and treat HDPs, and 3) poor communication of health results to clients, resulting in low maternal health literacy. These issues are generally exacerbated in PHCs vs. hospitals due to lower level cadres of healthcare workers and inadequacy of funding for essential supplies, equipment, and infrastructure. Future research may wish to explore additional elements of the quality of ANC in Nigeria and how it impacts birth and postnatal outcomes. This includes outcomes of eclampsia seizures, likelihood and success of cesarean section following PE/E indication, days of hospitalization, and admission to intensive care.

## Additional files

Additional file 1: Table of the prevalence of each indicator listed under provider interpersonal skills and comparison of prevalence between PHCs and hospitals. (DOCX 13 kb)

Additional file 2: Linear regression table examining the effect of client demographic variables including age, SES, and education on client outcome scores. (DOCX 13 kb)

## Data Availability

The datasets from the current study are available from the corresponding author on reasonable request.

## Abbreviations

ANC: antenatal care
BP: blood pressure
CHEW: Community Health Extension Worker
CHO: Community Health Officer
HDP: Hypertensive Disorders in Pregnancy
HIV: human immunodeficiency virus
HT: hypertension
LGA: Local Government Authorities
MgSO_4_: magnesium sulfate
PE/E: pre-eclampsia/ eclampsia
PHC: primary healthcare center
PMTCT: prevention of mother to child transmission
QOC: quality of care
SES: socioeconomic status
STI: sexually transmitted infection
TB: tuberculosis
WHO: World Health Organization
