# Supplementary material for: Detecting and managing hypertensive disorders in pregnancy: a cross-sectional analysis of the quality of antenatal care in Nigeria
Source: BMC Health Serv Res. 2019 Jun 24;19:411. doi: 10.1186/s12913-019-4217-8 (PMC6591953; doi:10.1186/s12913-019-4217-8)
Supplement: Supplementary file 1 — Table of the prevalence of each indicator listed under provider interpersonal skills and comparison of prevalence between PHCs and hospitals. (DOCX 13 kb) [file 12913_2019_4217_MOESM1_ESM.docx]

Supplementary table 1 – Prevalence of each indicator within provider interpersonal skills

| **Provider interpersonal skills** | **Total**  **n (%)** | **Primary**  **n (%)** | **Secondary**  **n (%)** | **p-value** |
| --- | --- | --- | --- | --- |
| **Rapport** |  |  |  |  |
| used client's name | 124 (91.9) | 39 (97.5) | 85 (89.5) | 0.016 |
| greeted client in friendly manner | 122 (89.7) | 40 (100) | 82 (85.4) | 0.011 |
| requested client take a seat | 132 (97.8) | 40 (100) | 92 (96.8) | 0.555 |
| maintained audio privacy | 115 (85.2) | 27 (67.5) | 88 (92.6) | 0.000 |
| maintained visual privacy | 112 (83.0) | 26 (65.0) | 86 (90.5) | 0.000 |
| treated client with respect | 115 (86.5) | 38 (95.0) | 77 (82.8) | 0.059 |
| client felt comfortable asking Qs | 125 (94.7) | 40 (100) | 85 (92.4) | 0.073 |
| **Communication** |  |  |  |  |
| used words client could understand | 125 (92.6) | 39 (97.5) | 86 (90.5) | 0.158 |
| listened to client attentively | 128 (94.8) | 39 (97.5) | 89 (93.7) | 0.361 |
| inquired for need of other services | 66 (51.2) | 14 (35.9) | 52 (57.8) | 0.022 |
| answered/explained clients inquiry | 110 (86.6) | 35 (94.6) | 75 (83.3) | 0.090 |
