# Supplementary material for: Detecting and managing hypertensive disorders in pregnancy: a cross-sectional analysis of the quality of antenatal care in Nigeria
Source: BMC Health Serv Res. 2019 Jun 24;19:411. doi: 10.1186/s12913-019-4217-8 (PMC6591953; doi:10.1186/s12913-019-4217-8)
Supplement: Supplementary file 2 — Linear regression table examining the effect of client demographic variables including age, SES, and education on client outcome scores. (DOCX 13 kb) [file 12913_2019_4217_MOESM2_ESM.docx]

Supplementary table 2 – linear regression coefficients for outcome score

| **Client Outcome Linear Regression** | **Beta coefficient** | **Std. Error** | **Lower 95% confidence interval** | **Upper 95% confidence interval** | **P-value** |
| --- | --- | --- | --- | --- | --- |
| **(Constant)** | 7.18 | 1.83 | 3.42 | 10.95 | 0.001 |
| **Facility Type (ref= PHC)** | 1.01 | 0.75 | -0.53 | 2.54 | 0.189 |
| **Age in completed years** | 0.01 | 0.05 | -0.09 | 0.11 | 0.782 |
| **Socioeconomic Status**  **(ref= low)** |  |  |  |  |  |
| Medium | -1.16 | 0.61 | -2.82 | 0.10 | 0.071 |
| High | -1.42 | 0.67 | -3.42 | -0.03 | 0.045 |
| **Education (ref=none)** |  |  |  |  |  |
| Nursery | -0.42 | 0.71 | -1.88 | 1.03 | 0.556 |
| Primary | -0.37 | 1.05 | -2.52 | 1.78 | 0.727 |
| Secondary | -0.60 | 0.66 | -1.96 | 0.76 | 0.374 |
| Postsecondary | 0.22 | 0.76 | -1.34 | 1.78 | 0.776 |
